# Predicting Blood Pressure and Blood Pressure Variability in Spontaneous Intracerebral Hemorrhage in the Emergency Department Using Machine Learning

**DOI:** 10.3390/jcm14217800

**Published:** 2025-11-03

**Authors:** Emmeline Leggett, Abigail Kim, Shriya Jaddu, Priya Patel, Nahom Y. Seyoum, Manahel Zahid, Angie Chan, Hassan Syed, Milana Shapsay, David Dreizin, Joshua Olexa, Jennifer A. Walker, Stephanie Cardona, Quincy K. Tran

**Affiliations:** 1 School of Medicine, University of Maryland at Baltimore, Baltimore, MD 20201, USA; emmeline.leggett@som.umaryland.edu (E.L.); nahoms1995@gmail.com (N.Y.S.); manahel.zahid@som.umaryland.edu (M.Z.); hassanksyed23@gmail.com (H.S.); 2 Research Associate Program in Emergency Medicine & Critical Care, Department of Emergency Medicine, University of Maryland School of Medicine, Baltimore, MD 20201, USA; kimay00@gmail.com (A.K.); shriyajaddu@gmail.com (S.J.); angiechan0127@gmail.com (A.C.); mshapsay@gmail.com (M.S.); jennifer.walker@bswhealth.org (J.A.W.); 3 Department of Emergency Medicine, University of Maryland School of Medicine, Baltimore, MD 20201, USA; pspatel@som.umaryland.edu; 4 Department of Diagnostic Radiology and Nuclear Imaging, Division of Trauma and Emergency Radiology, University of Maryland School of Medicine, Baltimore, MD 20201, USA; ddreizin@umm.edu; 5 Department of Neurosurgery, University of Maryland School of Medicine, Baltimore, MD 20201, USA; jolexa@som.umaryland.edu; 6 Department of Emergency Medicine, Baylor Scott & White All Saints Medical Center, Fort Worth, TX 76104, USA; 7 Department of Emergency Medicine, Burnett School of Medicine, Texas Christian University, Fort Worth, TX 76109, USA; 8 Department of Critical Care Medicine, Baptist Health System, Miami, FL 33143, USA; drscard013@gmail.com; 9 Program in Trauma, The R Adam Cowley Shock Trauma Center, University of Maryland School of Medicine, Baltimore, MD 20201, USA

**Keywords:** spontaneous intracerebral hemorrhage, blood pressure management, machine learning, blood pressure variability

## Abstract

**Introduction**: Spontaneous intracerebral hemorrhage (sICH) is a devastating type of stroke. Blood pressure reduction is crucial in its management and is well mentioned in current guidelines; however, the role of blood pressure variability (BPV) in emergency departments (EDs) has not been well studied. This study aimed to identify predictors of lower systolic blood pressure (SBP) (≤160 mmHg) and BPV at ED discharge and course, respectively. **Methods**: This is a retrospective study of prospectively collected data at a quaternary care center of adult patients diagnosed and treated with sICH between 1 January 2017 and 31 December 2020. The primary outcome of interest was SBP at ED discharge; this was divided into two groups: a control group composed of patients discharged with an SBP ≤ 160 mmHg and a comparison group composed of patients discharged with an SBP > 160 mmHg. Secondary outcomes included measures of BPV, specifically successive variation (SBPSV), and standard deviation (SBPSD) during ED course. Machine learning algorithms were used to identify predictors of SBP at ED discharge: SBPSV and SBPSV. **Results**: This study evaluated 142 patients, of which 85 (60%) were discharged with SBP ≤ 160 mmHg. The mean SBP at ED discharge was 133 (±16.1) mmHg for the control group and 184 (±21.3) for the comparison group (difference −51; 95% CI −58 to −45; *p* < 0.001). The top five predictors for the primary outcome identified by machine learning included initial SBP at ED triage, serum sodium, clevidipine administration, serum glucose, and serum creatinine. Predictors for secondary outcome included mechanical ventilation, serum glucose, and initial SBP at ED triage. **Conclusion**: Initial SBP was the top predictor of achieving a goal SBP ≤160 mmHg at ED discharge in patients with sICH. Predictors of increased BPV included mechanical ventilation, elevated serum glucose, and high initial SBP in the ED. While further studies are necessary to confirm our observations, clinicians should consider these factors when they care for patients with sICH.

## 1. Introduction

Spontaneous intracerebral hemorrhage (sICH) is defined as the non-traumatic extravasation of blood into brain parenchyma from a ruptured cerebral blood vessel [1]. It is the second most common type of stroke and also the deadliest, with estimated mortality rates of 35–52% at one month, with over half of these deaths occurring in the first two days [2]. Survivors are often left with substantial disabilities [3,4]. Over the past two decades, the annual incidence of symptomatic sICH has doubled, with approximately 80,000 occurrences per year. While blood pressure control has reduced the incidence in some populations, the absolute number of cases has increased, most likely due to the use of anticoagulation and an aging population [5]. Accordingly, it is imperative that we understand how to reduce the morbidity and mortality of this condition, as it will become increasingly prevalent in the coming years.

From the early 2000s to approximately 2017, the American Heart Association (AHA) and the American College of Cardiology (ACC) recommended lowering the blood pressure in patients with sICH to less than 160 mmHg. These recommendations were largely based on observational data and smaller trials, which showed a risk of cerebral hypoperfusion when blood pressure was lowered below 160 mmHg. In 2017, the AHA and ACC guidelines were updated following evidence from large randomized controlled trials, INTERACT1, INTERACT2, and ATACH2, which demonstrated that an acute reduction in SBP to <140 mmHg was associated with reduced hematoma expansion and improved functional outcomes, without an increase in adverse events [6,7]. However, subsequent data from the INTERACT3 trial indicated that lowering SBP further, to <130 mmHg, may be associated with worse outcomes. Consequently, although the current guidelines recommend more aggressive BP lowering, in clinical practice, many clinicians continue to target an SBP of 140–160 mmHg, a range that balances the potential benefits of hematoma control with the risk of cerebral hypoperfusion and aligns with long-standing clinical experience.

In addition to BP management, the AHA also recommends limiting BPV and achieving a smooth and sustained blood pressure, since this appears to reduce hematoma expansion and leads to improved neurologic outcomes [6,7]. It is hypothesized that increased blood pressure variability (BPV), in the initial 48 h window, subjects patients to high mortality rates and poor neurologic outcomes such as a lower Glasgow Coma Score. BPV is defined as the average of absolute differences between consecutive blood pressure measurements or variations in blood pressure during a period of time [8,9]. In particular, it has been shown that BPV with elevated systolic peaks is an independent predictor of poor outcomes in sICH [9]. Current guidelines on how to limit these systolic peaks, and further identifying what the target systolic range should be, remains controversial.

The aim of this study was to identify predictors of blood pressure at ED discharge and predictors of lower BPV during the ED course. We hypothesized that patients discharged from the ED with an SBP ≤ 160 mmHg had lower BPV during their ED course.

## 2. Methods

### 2.1. Study Design and Patient Selection

Patients’ data were retrospectively collected from a limited dataset that was prospectively maintained by members of our institution’s Department of Neurosurgery as part of routine clinical care for patients diagnosed with sICH. The study institution is a quaternary care center with neurosurgery coverage 24 h a day, seven days per week. When patients arrive at our institution, either from another facility or from our own ED, they are evaluated immediately by the neurosurgical team for interventions. After neurosurgery assessment, they typically undergo a second Computer Tomography (CT) scan within 6 to 25 h after admission to assess the stability of their hemorrhage.

All adult patients (age ≥ 18 years) who were treated at our institution for sICH between 1 January 2017 and 31 December 2020 were eligible. Patients were excluded if they were not accompanied with any ED records about their management while being in the ED prior to transferring to our institution, or if their hemorrhage was due to trauma, malignancy, arteriovenous malformations, ischemic stroke, or subarachnoid hemorrhage, as the underlying pathophysiology and clinical outcomes differ from those of sICH. Additionally, patients with non-intracerebral hemorrhages were excluded.

The study protocol was reviewed and approved by the Principal Investigator’s Institutional Review Board (HP-00084554). The requirement for informed consent was waived by the IRB due to the retrospective, observational nature of the study.

### 2.2. Outcome Measures

The primary outcome of the study was SBP at ED discharge, defined as the final recorded SBP value prior to the patient leaving the ED. The primary outcome was divided into two groups: SBP ≤ 160 mmHg which was the control group and SBP > 160 mmHg which was the comparison group. Secondary outcomes included measures of BPV, specifically the successive variation in SBP (SBPSV) and the standard deviation in SBP (SBPSD), during their ED stay.

### 2.3. Blood Pressure Variability

Since this was a retrospective study, blood pressure cuff placement and frequency was not standardized. Generally, blood pressure is obtained by an automated blood pressure cuff applied by a registered nurse (RN) in the ED and values are either transferred directly to the electronic medical record via device integration or manually entered by the patient’s RN. Because the frequency of blood pressure measurements was not standardized, we captured all recorded blood pressure measurements in the ED, from triage to ED discharge. Calculations for blood pressure variability were conducted, using the values reported in patients’ records. The standard deviation of SBP is a measure of how “tight” a patient’s SBP is, and is defined as the average of differences between one blood pressure measurement and the mean of those blood pressure measurements. Successive variation in SBP is the measure of how “quick” a patients’ SBP is reduced, and is defined as the average of absolute differences in two consecutive blood pressure measurements. The formulas to calculate SBPSD and SBPSV are as follows [8,9].

Successive variations in systolic blood pressure (SBPSV) were calculated as in Formula (1):

SBPSV = √(1(*n* − 1))∫(*SBPi* + 1 − *SBPi*)2(*n* − 1) (*i* = 1). (1)

Similarly, standard deviation in systolic blood pressure (SBPSD) was calculated as in Formula (2) [8]:

SBPSD = √(1*n*)∫(*BPi* − *BPmean*)2(*n* − 1) (*i* = 1). (2)

### 2.4. Data Collection and Management

Prior to data collection, investigators who were not blinded to the study hypothesis underwent training in data extraction under the supervision of the senior investigator. Training involved reviewing sets of five patient charts until a minimum inter-rater agreement of 90% was achieved.

Following training, data were extracted into a standardized Microsoft Excel spreadsheet (Microsoft Corp., Seattle, WA, USA). To reduce potential bias, data extraction responsibilities were divided such that investigators collecting information on interventions did not also extract blood pressure or outcome data, and vice versa. To enhance data reliability, a second investigator independently reviewed 20% of the dataset during the data collection phase to ensure continued inter-rater agreement of at least 90%. Missing data was inputted as normal values.

Data were primarily obtained from electronic medical records accessed through the Epic system (Epic Systems Corp., Verona, WI, USA) at the study institution, as well as from a limited neurosurgical dataset that had been prospectively maintained as part of routine clinical care by the team. The limited neurosurgical dataset included patients’ demographic information, types and location of hemorrhage, whether patients take antiplatelet or anticoagulation medication at home, and opening pressure if an external ventricular drain was inserted. For patients transferred from other hospitals’ emergency departments that did not use Epic, paper records that accompanied patients were reviewed.

A range of clinical variables were extracted from the medical record. Baseline information included past medical history and home medications. From the emergency department (ED) encounter, we collected data on blood pressure measurements, the presence of seizures, and the use of mechanical ventilation. We collected common laboratory values (sodium, glucose, and creatinine) that have been shown to be relevant to outcomes for patients of sICH and are readily available to Emergency Medicine clinicians. We imputed any missing data as a normal value. Components of the intracerebral hemorrhage (ICH) score were extracted, including age, ICH volume, the presence of intraventricular hemorrhage, and infratentorial hemorrhage. The ICH volume was calculated according to the ABC/2 method by our Radiology team, which included a Radiology attending and a resident. Any discrepancies were resolved by discussion between members of the team [10,11]. Furthermore, ED therapies, including antihypertensive medications, anti-epileptic medications, blood products, and intravenous crystalloids were also extracted. Medications that were ordered but not administered in the ED were not included.

### 2.5. Statistical Analysis

We based our sample size calculation on a previous study that observed a reduction of 30 mmHg in SBP between ED triage and leaving ED, among patients with both sICH and subarachnoid hemorrhage [12]. Therefore, we planned to include a sample size of at least 48 patients per group, to detect a difference of 20 mmHg of SBP between 2 groups, with a standard deviation of 30 mmHg at leaving ED, to achieve a power of 90% and alpha of 0.05. Similarly, we estimated that we would need a total of 120 patients or 60 per group to detect a difference of 10 mmHg in BPV with a standard deviation of 20 mmHg.

Descriptive analyses were used to present the data. Prior to analyses, histograms for continuous variables were generated to determine their distributions. An additional Anderson–Darling’s test was performed to assess the compliance of the distribution of those quantitative variables that were reported to have normal distributions. The results of the Anderson–Darling’s tests are reported in the Appendix. Parametric data (normally distributed data) was analyzed by the Student *t*-test and presented as the mean (standard deviation [SD]). Non-parametric data was analyzed by the Mann–Whitney U test and presented as the median (interquartile range [IQR]). For categorical variables, Pearson’s chi square test was used to compare the percentages between groups. These comparisons are represented in the final results as differences between groups, 95% confidence interval, and *p*-value.

### 2.6. Machine Learning

Machine learning algorithms were developed to predict any factors that could be associated with patients who had SBP < 160 mmHg at ED discharge, as well as SBPSD and SBPSV. Due to potential collinearity, only the triage SBP values were included in the models predicting the corresponding outcomes. Patients with missing values for the target (outcome) variable were excluded from this analysis.

Prior to modeling our machine learning algorithms, a list of variables (Appendix A) was selected according to their perceived clinical importance to patients’ SBP.

The Random Forest (RF) and XGBoost models were implemented using SciKit-learn libraries (version 1.6.1), such that one model was implemented for each outcome. We planned to use XGBoost models as validation analyses to validate the predictors being identified by the RF models. As such, any predictors being identified in the top five predictors for the outcome of interest by both the RF and XGBoost models were considered significant predictors.

For the RF models, all remaining missing values in predictor variables were imputed as zero, given RF’s inability to handle null values. In contrast, for the XGBoost models, all missing values in predictor variables were left blank, and XGBoost’s mechanism for handling null values was leveraged. Classification models were used for the binary outcome of SBP < 160 mmHg, and regression models were used for SBPSD and SBPSV, both of which were continuous variables.

A five-fold cross-validated grid search approach was used to optimize important hyperparameters and for pruning each model. These hyperparameters included the number of estimators, maximum tree depth, learning rate, and subsample ratio. Final models were trained using the optimized hyperparameters and evaluated on a held-out testing dataset. For classification models of the binary outcome (SBP ≤ 160 mmHg vs. SBP > 160 mmHg), the accuracy and F1 scores were recorded. Accuracy represents the proportion of correct predictions, and the F1 score, the harmonic mean of precision and recall, is useful for evaluating models on datasets with imbalanced classes. Both F1 scores and accuracy range from 0 (no correct prediction) to 1 (perfect prediction). For regression models (SBPSD and SBPSV), the root mean squared error (RMSE) was recorded. RMSE reflects the square root of the average differences between predicted and actual values, so lower RMSE values indicate better prediction.

To understand feature importance, SHapley Additive exPlanations (SHAP) was employed following training and testing. SHAP utilizes a game theory approach to quantify a feature’s importance in a model’s prediction. SHAP was utilized to identify the five most influential predictors in each model as well as their corresponding mean absolute SHAP values. Larger mean absolute SHAP values indicate a larger influence on a model’s predictions (i.e., a significant predictor). SHAP summary plots were also generated to visualize relative feature importance.

We also used an additional model that was trained on only the top five predictors for each outcome, while keeping the hyperparameters and patient population constant as a sensitivity analysis. The accuracies and RMSE values of these models were compared to the accuracies and RMSE values of the original models to assess whether their top five predictors alone were indeed significant.

Descriptive analyses were performed using Minitab version 20 (Minitab, LLC, State College, PA, USA). All statistical analyses with *p*-value < 0.05 were considered statistically significant. Machine learning algorithms were modeled with Python version 3.12.11 (Python Software Foundation, www.python.org) and Google Colab (https://colab.research.google.com/). Pandas (version 2.2.2) and NumPy (version 2.0.2) were used for data preprocessing, and Scikit-Learn (version 1.6.1) was used for model building.

## 3. Results

### 3.1. Patient Characteristics

**Figure 1:**
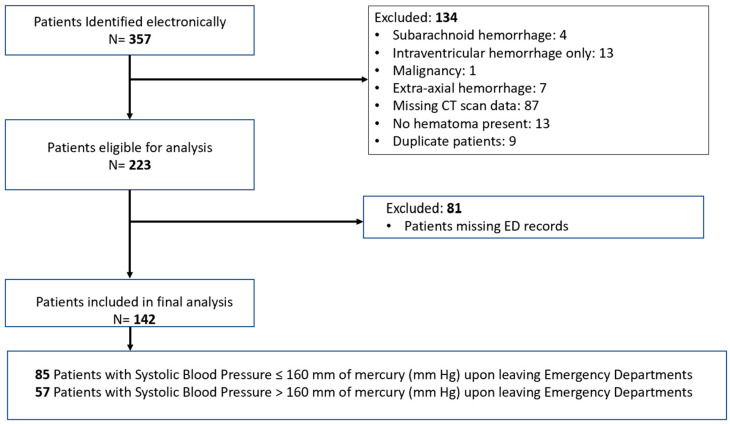
Patient selection diagram.

We identified and analyzed the charts of 142 patients who met our inclusion criteria (Figure 1). Among these patients, 85 (60%) were in the SBP ≤ 160 mmHg group (control group), while 57 (40%) were classified in the SBP > 160 mmHg group (comparison group). All blood pressure measurements were recorded via a non-invasive cuff blood pressure modality. The mean (±SD) age for the control group was 62.5 (±16.1) and for the comparison group was 62.7 (±12.7) (difference −0.24, 95% CI −5.04 to 4.57, *p* = 0.92) (Table 1).

A higher proportion (86%) of patients in the comparison group had a history of hypertension, with 24% more patients reporting a prior history of hypertension compared with the control group (62%) (*p* < 0.001). The mean initial ED triage SBP of the comparison group was 30 mmHg higher than the control group. Most of the other demographic variables were similar between both groups (Table 1).

Regarding treatment, there were more patients in the comparison group receiving crystalloids (difference 20%, 95% CI 6% to 33%, *p* = 0.01) and clevidipine infusion (difference 18%, 95% CI −0.33 to 0.06, *p* = 0.003) than the control group (Table 1). Most other treatments were similar between groups.

Although patients in the comparison group had a longer median ED length of stay (difference 57 min, 95% CI 26 to 88, *p* < 0.001) than the control group, their SBPSD and SBPSV were similar (Table 1).

### 3.2. Primary Outcome: Systolic Blood Pressure at ED Discharge

The mean SBP at ED discharge was 133.2 (±16.1) for the control group and 184.7 (±21.3) mmHg for the comparison group (difference −51.5, 95% CI −58 to −45, *p* < 0.001) (Table 1).

For the Random Forest classification outcome of whether the final SBP was ≤160 mmHg, the top five predictors identified by SHAP analysis were the initial SBP at ED triage (mean absolute SHAP value: 0.14), serum sodium (0.028), clevidipine administration (0.026), serum glucose (0.022), and serum creatinine (0.021) (Table 2). This model achieved a good overall accuracy of 72.4% and an F1 score of 0.76.

**Figure 2:**
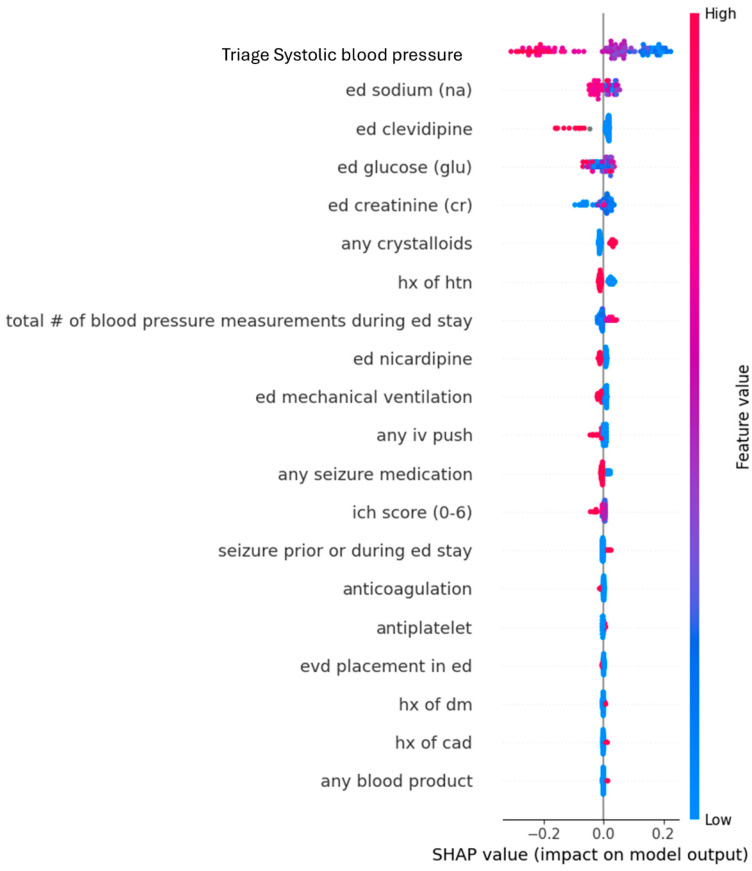
Dot plots from Random Forest analysis for features predicting SBP ≤ 160 mmHg at leaving emergency departments. The Y-axis represents the order of significant contributions from top (most significant) to bottom (least significant). The X-axis represents the Shapley Additive Explanations (SHAP) values. Red dots represent high values while blue dots represent low values. Dots extending toward the right of the center predict a higher likelihood of achieving the outcome of SBP ≤ 160 mmHg. Abbreviations: CAD, coronary artery disease; DM, diabetes mellitus; ED, Emergency Department; EVD, external ventricular drain; HTN, hypertension; Hx, history; ICH, intracerebral hemorrhage; IV, intravenous; SHAP, Shapley Additive Explanations.

From the Random Forest plot, a higher initial SBP at ED triage predicted a lower likelihood of patients achieving an SBP ≤ 160 mmHg when they were discharged from the referring ED to leave for the tertiary care center (Figure 2).

The XGBoost algorithm also predicted the same top five features as the Random Forest algorithm, although with a slightly higher accuracy and different order for the features (Table 2). Nonetheless, it confirms the results from the Random Forest algorithm.

### 3.3. Secondary Outcomes: Blood Pressure Variability

#### 3.3.1. Successive Variation in SBP (SBPSV)

**Figure 3:**
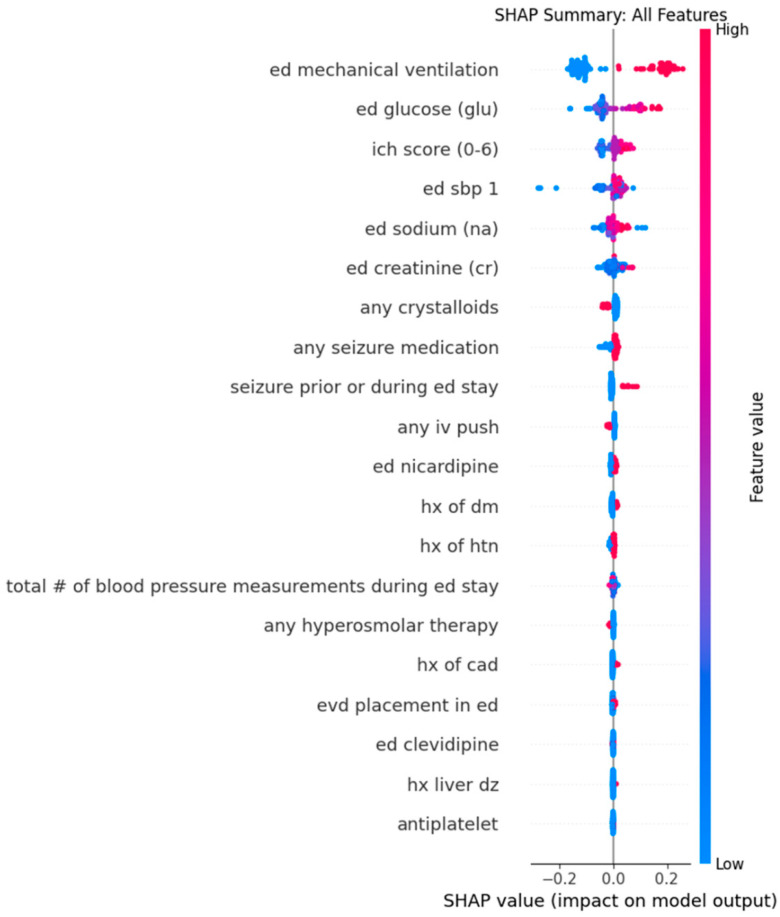
Dot plots from Random Forest analysis for features predicting Successive Variation in Systolic Blood Pressure during Emergency Department stay. The Y-axis represents the order of significant contributions from top (most significant) to bottom (least significant). The X-axis represents the Shapley Additive Explanations (SHAP) values. Red dots represent high values while blue dots represent low values. Dots extending toward the right of the center predict higher values for SBPSV. Abbreviations: CAD, coronary artery disease; DM, diabetes mellitus; dz, disease; ED, Emergency Department; EVD, external ventricular drain; HTN, hypertension; Hx, history; ICH, intracerebral hemorrhage; IV, intravenous; SBP, systolic blood pressure; SHAP, Shapley Additive Explanations.

**Figure 4:**
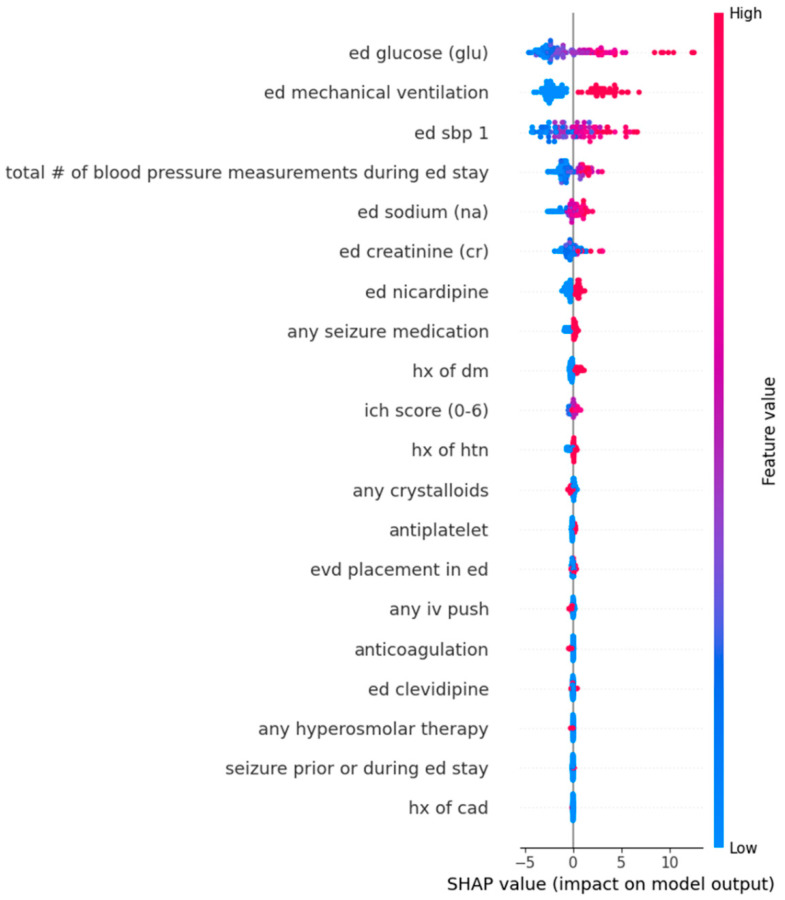
Dot plots from Random Forest analysis for features predicting Standard Deviation in Systolic Blood Pressure during Emergency Department stay. The Y-axis represents the order of significant contributions from top (most significant) to bottom (least significant). The X-axis represents the Shapley Additive Explanations (SHAP) values. Red dots represent high values while blue dots represent low values. Dots extending toward the right of the center predict higher values for SBPSD. Abbreviations: CAD, coronary artery disease; DM, diabetes mellitus; ED, Emergency Department; EVD, external ventricular drain; HTN, hypertension; Hx, history; ICH, intracerebral hemorrhage; IV, intravenous; SHAP, Shapley Additive Explanations.

For the continuous outcome of SBPSV, the Random Forest regression identified the top five predictors for SBPSV: ED mechanical ventilation (mean absolute SHAP value: 0.1458), serum glucose (0.0638), ICH score (0.0295), initial SBP measurement in the ED (0.0278), and serum sodium (0.0190). The model had a root mean squared error (RMSE) of 24.22 (Table 3). In the Random Forest dot plot, higher values for serum glucose predict higher SBPSV (Figure 3 and Figure 4) and lower serum glucose predicts lower SBPSV. Similarly, ED mechanical ventilation also predicted higher SBPSV.

For the sensitivity analysis, the XGBoost regression also identified the top five predictors for SBPSV: ED mechanical ventilation (mean absolute SHAP value: 0.1108), serum glucose (0.0298), the administration of any seizure medication (0.0293), serum sodium (0.0287), and serum creatinine (0.0175) (Table 2). The model had a root mean squared error (RMSE) of 24.95.

Therefore, both the Random Forest and XGBoost models agreed on ED mechanical ventilation, serum glucose (0.0638), and serum sodium as the top features that predicted SBPSV in the ED.

#### 3.3.2. Standard Deviation of SBP (SBPSD)

For the continuous outcome of SBPSD, the Random Forest model identified the top five predictors: serum glucose (mean absolute SHAP value: 2.7908), ED mechanical ventilation (2.6038), initial SBP measurement in the ED (2.0517), total number of blood pressure measurements during the ED stay (1.0598), and serum sodium (0.6671). The model had a root mean squared error (RMSE) of 13.66. From the Random Forest dot plot, higher serum glucose and ED mechanical ventilation predicted higher SBPSD (Table 4), (Figure 4).

Similarly, the XGBoost model was utilized for sensitivity analysis. This XGBoost model also predicted the top five predictors by SHAP analysis as serum glucose (mean absolute SHAP value: 2.9724), initial SBP measurement in the ED (2.3626), ED mechanical ventilation (2.2050), total number of blood pressure measurements during the ED stay (1.8989), and serum sodium (1.5057). The model had a root mean squared error (RMSE) of 14.46. All of these top five features were agreed upon by both the Random Forest and XGBoost models as predictors for SBPSD during patients’ ED stay.

## 4. Discussion

This study is a retrospective, single-center observational study investigating predictors of systolic blood pressure and blood pressure variability in patients with sICH in the ED. We identified that approximately 60% of the patients in our study achieved an SBP ≤ 160 mmHg upon ED discharge. Both the Random Forest and XGBoost models, with good accuracy, identified a few common features that predicted achieving SBP ≤ 160 mmHg at ED discharge, and predicted SBPSV and SBPSD during ED stay. Initial SBP in ED triage, serum sodium, glucose, and creatinine, and the use of clevidipine predicted an SBP ≤ 160 mmHg upon ED discharge. Mechanical ventilation, values of serum glucose, and initial SBP measurement in the ED were predictors of SBPSV and SBPSD during ED stay. Accordingly, each of these factors should serve as indicators for hypervigilance among ED staff when treating patients with sICH.

Our study identified that initial SBP in ED triage, serum sodium, glucose, and creatinine levels, and the use of clevidipine predicted an SBP ≤ 160 mmHg upon ED discharge. While it is intuitive that patients who presented with a higher SBP were less likely to achieve desired BP control to ≤160 mmHg, this result highlights the complexity of SBP management in patients with ICH. Achieving optimal control requires balancing the risks of both insufficient and excessive reduction. Excessively aggressive lowering can precipitate adverse events, as illustrated by the ATACH-2 trial, in which 1000 patients with acute ICH randomized to intensive treatment (target SBP 110–139 mmHg) versus standard of care (140–179 mmHg) experienced a higher incidence of renal adverse events compared with the standard treatment group [7]. Similarly, in INTERACT2, which enrolled 2839 patients, a rapid reduction to below 130 mmHg was associated with worse neurological outcomes [13]. Conversely, an inadequate or delayed reduction may permit hematoma expansion, neurologic deterioration, and poorer functional recovery, as demonstrated in the INTERACT 1–4 trials [14]. Together, these data emphasize that blood pressure management in ICH is a dynamic process requiring careful titration to minimize both ischemic and hemorrhagic risks.

The use of clevidipine as a predictor of SBP ≤ 160 mmHg is clinically relevant as it provides insight into best practices for emergency medicine clinicians. The previous literature has concluded that both clevidipine and nicardipine are equally effective at lowering the net SBP values to the target range [15,16]. Additionally, the early initiation of nicardipine in the ED for patients with spontaneous intracranial hemorrhage was also associated with a lower rate of acute kidney injury. Our study further solidifies the recommendations on the use of these agents for fast, smooth, and adequate blood pressure control [17].

A higher serum sodium, blood glucose, and lower creatinine levels were all negative predictors of SBP ≤ 160 mmHg. It has been shown that there is a correlation between high serum sodium concentrations and increased cardiovascular risk in hypertensive individuals. The leading theories are that higher sodium may exert direct vascular effects in hypertensive patients, such as dysregulating the tissue renin–angiotensin response [18,19]. Similarly, there is a known correlation between uncontrolled diabetes and hypertension, with many pathophysiological links [20]. Perhaps most relevant to our findings is that diabetes mellitus results in enhanced sodium transport via the enhanced activity of the sodium–hydrogen exchanger-3 and thus fluid retention, contributing to both hypertension and increased sodium [21]. Likewise, uncontrolled diabetes is associated with decreased muscle mass, which could explain the inverse relationship between serum creatinine and SBP [22].

Regarding our secondary outcome, BPV, we determined that mechanical ventilation, increased serum glucose, and increased serum sodium were all predictors of both increased SBPSV and SBPSD. Additionally, a higher initial SBP measurement in the ED and the greater total number of SBP measurements during the ED stay were also associated with increased SBPSD. In a pooled analysis of the INTERACT2 and ATACH-II trials, it was shown that an early reduction in BPV correlates to more favorable outcomes [23]. A possible explanation for the correlation between mechanical ventilation and BPV could be related to the need for sedation and analgesia. Insufficient analgesia or sedation in patients undergoing intubation, especially in patients with sICH, is associated with agitation and consequent fluctuations in blood pressures [24]. Accordingly, our finding aligns with a recent study that highlights the need for pain control in addition to antihypertensives in patients with ICH [25]. Therefore, we strongly recommend emergency medicine clinicians to initiate early sedation and pain management for patients who have sICH and require invasive mechanical ventilation for patients’ comfort, and a reduction in BPV post intubation. Given that increased BPV is associated with poor neurological outcomes in patients with ICH, our study also emphasizes the need for further investigation into more effective antihypertensives that both control SBP and BPV, even upon leaving the ED [9].

Our study’s correlation between blood glucose and BPV has been well described in the literature as it has been shown that patients with diabetes have a higher variability of daytime BP than patients without diabetes [26]. One hypothesis for this correlation could be the relationship between diabetes and cardiovascular autonomic neuropathy—hyperglycemia induces inflammation which damages nerve fibers, disrupting sympatho-vagal regulation [27]. The relationship between serum sodium and BPV, on the other hand, has been less studied. The previous literature demonstrates that serum sodium variability is correlated with increased BPV and worse clinical outcomes in ICH patients, yet their hypothesis was with regard to fluid management [28]. Our finding of increased serum sodium as a predictor of BPV, therefore, serves as a potential area for further exploration. One hypothesis previously alluded to is that serum sodium may have direct vascular effects in hypertensive patients, similar to those of glucose, which may dysregulate autonomic function or vascular response [18]. Interestingly, hyperosmolar therapy (mannitol and hypertonic saline) was not found to be a top predictor for SBP at ED discharge or BPV, and was not statistically different between patients who achieved an SBP ≤ 160 mmHg and those who did not. Although hyperosmolar therapy decreases ICP by creating an osmotic gradient and drawing water out of the brain parenchyma, its effect on systemic blood pressure is only transient and is a result of the transient increase in cardiac output and sympathetic system activation [29,30]. Similarly, the presence of an extra ventricular drain, which also decreases ICP by draining cerebrospinal fluid, was not associated with our primary outcome of interest. While it might be expected that reductions in ICP would elicit systemic blood pressure changes to maintain cerebral perfusion pressure, ICP alterations generally occur gradually and therefore produce only mild and short-lived effects on systemic blood pressure [31].

### Limitations

Our study has several limitations. First, this is a single-center study at a large academic center with 24 h and 7 days a week coverage by in-hospital neurosurgical services. These results might not apply to other centers or other environments of care with different patient populations or available consulting services. This was also a retrospective analysis of a dataset, and while efforts were made to reduce potential bias and to increase data reliability in data collection, errors in the record or in data imputation are possible. Some data were not available, and these data were imputed as normal values in most of our statistical analyses, except in the XGBoost algorithm which handles missing data through its own algorithm. Similarly, although we performed five-fold cross-validated pruning of our models, and were able to achieve good overall accuracy, these features may not predict well in other patient populations, due to the small sample size.

Furthermore, although this study captured whether patients received treatment during their ED stay, information regarding dosages and the timing of administration was not available. Consequently, we were unable to determine which dosing strategies or timing of clevidipine were most effective in achieving an SBP ≤ 160 mmHg at ED discharge. All blood pressure measurements were obtained using non-invasive cuff monitoring, which may not accurately reflect values obtained through invasive arterial monitoring. In addition, we did not specify whether blood pressure recordings were taken from the left or right arm or at what frequency the blood pressure was taken, which could introduce variability in certain patients.

Lastly, we did not capture all possible biomarkers that may correlate with clinical outcomes. As an example, troponin levels have been associated with increased mortality in patients with sICH [32,33]. Because troponin levels are not routinely obtained in the emergency department for this population, we were unable to assess potential associations with SBP and/or BPV. This represents an area for future investigation.

## 5. Conclusions

In summary, our study identified initial ED SBP as the strongest predictor of achieving a target SBP ≤ 160 mmHg in patients with sICH. Mechanical ventilation, elevated serum glucose, and higher initial blood pressure were key determinants of increased BPV. Given that both elevated SBP and greater BPV are associated with worse neurological outcomes, these findings highlight the importance of the early recognition and targeted management of these predictors in the acute care setting. Further research is warranted to validate these associations and guide evidence-based strategies for optimizing blood pressure control in sICH.

## Figures and Tables

**Table 1:** Demographic information for 142 patients with spontaneous intracerebral hemorrhage.

Parameters	All Patients	SBP ≤ 160 mmHg	SBP > 160 mmHg	Difference Between Group	95% CI	*p*
	N = 142	N = 85 (60%)	N = 57 (40%)			
**Demographics**						
Age, years, mean (SD)	62.6 (14.8)	62.5 (16.1)	62.7 (12.7)	−0.24	(−5.04, 4.57)	0.92
Age ≥ 80 years, N (%)	19 (13.4)	13 (15.3)	6 (10.5)	0.05	(−0.06, 0.16)	0.4
**Past medical history, N (%)**						
Coronary Artery Disease	15 (10.6)	11 (12.9)	4 (7)	0.06	(−0.04, 0.16)	0.4
Diabetes Mellitus	37 (26.1)	22 (25.9)	15 (26.3)	−0.004	(−0.15, 0.14)	0.95
Hypertension	102 (71.8)	53 (62.4)	49 (86)	−0.24	(−0.37, −0.10)	0.001
Chronic Kidney Disease	13 (9.2)	9 (10.6)	4 (7)	0.04	(−0.06, 0.13)	0.56
Any liver disease	2 (1.4)	2 (2.4)	0 (0)	0.02	(−0.01, 0.06)	0.52
**Past medications, N (%)**						
Any antiplatelet	39 (27.5)	22 (25.9)	17 (29.8)	−0.04	(−0.19, 0.11)	0.61
Any anticoagulation	22 (15.5)	14 (16.5)	8 (14)	0.02	(−0.10, 0.14)	0.69
**Clinical characteristics upon ED admission**						
Initial ED SBP (mmHg), mean (SD)	173.6 (37.5)	161.6 (33.1)	191.4 (36.7)	−30	(−41.83, −17.89)	<0.001
Initial ED heart rate (bpm), mean (SD)	85.8 (22.3)	86.9 (21.9)	83.9 (23.3)	3	(−5.28, 11.25)	0.48
Sodium (mEq/L), mean (SD)	139.1 (3.6)	139.1 (3.6)	139.2 (3.7)	−0.06	(−1.29, 1.17)	0.93
Creatinine (mg/dL), median [IQR]	1 [0.8–1.2]	0.9 [0.8–1.2]	1 [0.8–1.3]	−0.1	(−0.20, 0.02)	0.13
Glucose (mg/dL), median [IQR]	139.5 [118–177.3]	140 [119–177]	137 [115–177.5]	1	(−14, 15)	0.92
**Other clinical characteristics**						
GCS on ED admission, median [IQR]	13 [8.8–15]	13 [9.5–15]	13 [7–15]	0	(0, 2)	0.15
Presenting parenchymal hematoma volume (mL), median [IQR]	19 [7.3–35.9]	16 [7.5–34]	21.3 [7–39.4]	−1.71	(−7.82, 3.25)	0.5
ICH volume ≥ 30 mL, N (%)	50 (35.2)	28 (32.9)	22 (38.6)	−0.06	(−0.22, 0.10)	0.49
Intraventricular hemorrhage, N (%)	83 (58.5)	42 (49.4)	41 (71.9)	−0.23	(−0.38, −0.07)	0.01
Infratentorial bleed, N (%)	27 (19)	15 (17.6)	12 (21.1)	−0.03	(−0.17, 0.10)	0.62
ICH score, mean (SD)	1.8 (1.1)	1.7 (1.0)	2.1 (1.2)	−0.38	(−0.76, −0.002)	0.05
Clinical seizure prior or during ED stay, N (%)	15 (10.6)	11 (12.9)	4 (7)	0.06	(−0.04, 0.16)	0.4
Mechanical ventilation, N (%)	57 (40.1)	31 (36.5)	26 (45.6)	−0.09	(−0.26, 0.07)	0.28
Interval of triage to ED mechanical ventilation (minutes), median [IQR]	63 [36.5–116.5]	67 [33–149]	57.5 [41.5–94]	14	(−13, 48)	0.26
EVD placement in ED, N (%)	44 (31)	25 (29.4)	19 (33.3)	−0.04	(−0.20, 0.12)	0.62
ED Length of stay (minutes), median [IQR]	174.5 [129.8–269.8]	207 [150–286]	146 [103.5–220.5]	57	(26, 88)	<0.001
SBP at Leaving ED (mmHg), mean (SD)	153.9 (31.2)	133.2 (16.1)	184.7 (21.3)	−51.5	(−58, −45)	<0.001
Mortality (Hospice/Death), N (%)	31 (21.8)	18 (21.2)	13 (22.8)	−0.02	(−0.16, 0.12)	0.82
**Medical therapy**						
Any crystalloids, N (%)	39 (27.5)	30 (35.3)	9 (15.8)	0.2	(0.06, 0.33)	0.01
Any Nicardipine, N (%)	63 (44.4)	32 (37.6)	31 (54.4)	−0.17	(−0.33, −0.002)	0.05
Interval of triage to ED Nicardipine infusion (minutes), median [IQR]	68 [33–133.5]	104 [36–144.5]	56 [22–99.5]	27	(−1, 66)	0.06
Any Clevidipine, N (%)	17 (12)	4 (4.7)	13 (22.8)	−0.18	(−0.30, −0.06)	0.003
Interval of triage to ED Clevidipine infusion (minutes), median [IQR]	45 [27.5–76.5]	59.5 [42.8–198.5]	32 [22–69.5]	22	(−18, 182)	0.23
Both Nicardipine and Clevidipine, N (%)	1 (0.7)	1 (1.2)	0 (0)	0.01	(−0.01, 0.03)	0.99
**Any IV push antihypertensive, N (%)**	31 (21.8)	16 (18.8)	15 (26.3)	−0.07	(−0.22, 0.07)	0.3
>1 IV push, N (%)	4 (2.8)	2 (2.4)	2 (3.5)	−0.01	(−0.07, 0.05)	0.99
Interval of triage to first ED IV push (minutes), median [IQR]	66 [33–109]	74 [36.8–104.5]	58 [25–120]	6	(−38, 47)	0.76
Interval of triage to second ED IV push (minutes), median [IQR]	278 [199.3–1431.5]	1064 [329–1799]	208.5 [190–227]	856	(102, 1609)	0.25
**Any seizure medication, N (%)**	99 (69.7)	57 (67.1)	42 (73.7)	−0.07	(−0.22, 0.09)	0.39
Any Phenytoin, N (%)	4 (2.8)	4 (4.7)	0 (0)	0.05	(0.002, 0.09)	0.15
Any Levetiracetam/Keppra, N (%)	96 (67.6)	54 (63.5)	42 (73.7)	−0.1	(−0.25, 0.05)	0.2
>1 seizure medication, N (%)	1 (0.7)	1 (1.2)	0 (0)	0.01	(−0.01, 0.03)	0.99
**Any hyperosmolar therapy, N (%)**	21 (14.8)	13 (15.3)	8 (14)	0.01	(−0.11, 0.13)	0.84
3% saline, N (%)	2 (1.4)	2 (2.4)	0 (0)	0.02	(−0.01, 0.06)	0.52
Mannitol, N (%)	19 (13.4)	11 (12.9)	8 (14)	−0.01	(−0.13, 0.10)	0.85
**Any blood product, N (%)**	12 (8.5)	7 (8.2)	5 (8.8)	−0.01	(−0.10, 0.09)	0.91
Fresh frozen plasma, N (%)	2 (1.4)	1 (1.2)	1 (1.8)	−0.01	(−0.05, 0.04)	0.99
Platelets, N (%)	3 (2.1)	1 (1.2)	2 (3.5)	−0.02	(−0.08, 0.03)	0.56
PCC, N (%)	9 (6.3)	6 (7.1)	3 (5.3)	0.02	(−0.06, 0.10)	0.74
**Blood pressure variability**						
SBP standard deviation, median [IQR]	18.7 [12.1–26]	16.3 [11–25.6]	20.2 [13.9–27.9]	−3.31	(−6.74, 0.08)	0.05
SBP successive variation, median [IQR]	18.4 [12.5–25.9]	18.1 [11.2–23.5]	21 [14.4–29.9]	−3.18	(−6.60, 0.04)	0.05
**Heart rate variability**	N = 132	N = 80	N = 52			
Heart rate standard deviation, median [IQR]	7.9 [4.7–12.2]	7.7 [4.1–10.6]	8.6 [5.4–12.7]	−1.37	(−3.35, 0.43)	0.15
Heart rate successive variation, median [IQR]	8.5 [4.6–12.9]	7.8 [4.4–11.7]	10.2 [5.1–13.5]	−1.32	(−3.33, 0.84)	0.19
**Hematoma progression**						
Hematoma Volume Change Score ≥ 30%, N (%)	40 (28.2)	20 (23.5)	20 (35.1)	−0.12	(−0.27, 0.04)	0.14
Absolute Hematoma Change Score ≥ 12.5 mL, N (%)	20 (14.1)	11 (12.9)	9 (15.8)	−0.03	(−0.15, 0.09)	0.64

Missing creatinine values for 11 patients were imputed as 1.0 mg/dL, and missing glucose values for 4 patients as 100 mg/dL. Abbreviations: CI, confidence Interval; ED, Emergency Department; EVD, external ventricular drain; GCS, Glascow Coma Scale; ICH, intracerebral hemorrhage; IQR, interquartile range; IV, intravenous; PCC, prothrombin Complex Concentrate; SBP, systolic blood pressure; SD, standard deviation.

**Table 2:** Results from Random Forest and XGBoost algorithms for top five predictors of patients’ achieving systolic blood pressure ≤160 mmHg when patients left emergency departments.

	Outcome: SBP < 160 at Leaving ED	
	Random Forest Classification	XGBoost Classification
Accuracy	72.40%	79.30%
F1 score	0.76	0.79
Feature 1 (mean absolute SHAP value)	Triage SBP (0.14)	Triage SBP (0.13)
Feature 2 (mean absolute SHAP value)	Serum sodium (0.028)	Serum Sodium (0.034)
Feature 3 (mean absolute SHAP value)	Clevidipine (0.026)	Serum Glucose (0.029)
Feature 4 (mean absolute SHAP value)	Serum Glucose (0.022)	Clevidipine (0.027)
Feature 5 (mean absolute SHAP value)	Serum Creatinine (0.021)	Serum Creatinine (0.026)

ED, Emergency Department; SBP, systolic blood pressure; SHAP, Shapley Additive Explanations.

**Table 3:** Results from Random Forest and XGBoost algorithms for top five predictors of Successive Variation in Systolic Blood Pressure (SBPSV) when patients left emergency departments.

	Outcome: Successive Variation in Systolic Blood Pressure During ED	
	Random Forest	XGBoost
Root mean squared error (RMSE) *	24.21	24.94
Feature 1 (mean absolute SHAP value)	Mechanical ventilation in ED (0.15)	Mechanical ventilation in ED (0.11)
Feature 2 (mean absolute SHAP value)	Serum Glucose (0.064)	Serum Sodium (0.030)
Feature 3 (mean absolute SHAP value)	ICH score (0.029)	Any seizure medication (0.029)
Feature 4 (mean absolute SHAP value)	Triage SBP (0.027)	Serum Sodium (0.028)
Feature 5 (mean absolute SHAP value)	Serum Sodium (0.019)	Serum Creatinine (0.017)

* Lower RMSE value indicates better fit models. EDs, Emergency Departments; ICH, intracerebral hemorrhage score; SBP, systolic blood pressure; SHAP, Shapley Additive Explanations.

**Table 4:** Results from Random Forest and XGBoost algorithms for top five predictors of Standard Deviation in Systolic Blood Pressure (SBPSD) when patients left emergency departments.

	Outcome: Standard Deviation in Systolic Blood Pressure During ED	
	Random Forest	XGBoost
Root mean squared error (RMSE) *	13.66	14.46
Feature 1 (mean absolute SHAP value)	Serum Glucose (2.79)	Serum Glucose (2.84)
Feature 2 (mean absolute SHAP value)	Mechanical ventilation in ED (2.60)	Mechanical ventilation in ED (2.56)
Feature 3 (mean absolute SHAP value)	Triage SBP (2.05)	Triage SBP (2.36)
Feature 4 (mean absolute SHAP value)	Total number of BP Measurements (1.06)	Total number of BP Measurements (1.12)
Feature 5 (mean absolute SHAP value)	Serum Sodium (0.66)	Serum Sodium (0.64)

* Lower RMSE value indicates better fit models. Abbreviations: BP, blood pressure; EDs, Emergency Departments; SBP, systolic blood pressure; SHAP, Shapley Additive Explanations.

## Data Availability

The datasets presented in this article are not readily available because of restrictions from our Institutional Review Board.

## Appendix A. List of Variables Included in Random Forest and XGBoost Models (In the Order as They Appeared in Table 1)

History of CAD.

History of DM.

History of HTN.

History of CKD.

History of liver disease.

Antiplatelet medication.

Anticoagulation medication.

Serum sodium (Na).

Serum creatinine (Cr).

Serum glucose (Glu).

Intracerebral hemorrhage score (ICH).

Seizure prior or during ED stay.

Invasive mechanical ventilation.

EVD placement.

Any crystalloids.

Nicardipine infusion.

Clevidipine infusion.

Both nicardipine and clevidipine.

Any intravenous push antihypertensive medication.

Any seizure medication.

Any hyperosmolar therapy.

Any blood product.

Total number of ED systolic blood pressure (SBP) measurements.

ED triage SBP.

## Appendix B. Dot Plots from XGBoost Analysis for Features Predicting SBP ≤ 160 mmHg upon Leaving Emergency Departments

The Y-axis represents the order of significant contributions from top (most significant) to bottom (least significant). The X-axis represents the Shapley Additive Explanations (SHAP) values. Red dots represent high values while blue dots represent low values. Dots extending toward the right of the center predict a higher likelihood of achieving the outcome of SBP ≤ 160 mmHg.

Abbreviations: CAD, coronary artery disease; DM, diabetes mellitus; ED, Emergency Department; EVD, external ventricular drain; HTN, hypertension; Hx, history; ICH, intracerebral hemorrhage; IV, intravenous; SHAP, Shapley Additive Explanations; CKD, chronic kidney disease.

## Appendix C. Dot Plots from XGBoost Analysis for Features Predicting Successive Variation in Systolic Blood Pressure During Emergency Department Stay

The Y-axis represents the order of significant contributions from top (most significant) to bottom (least significant). The X-axis represents the Shapley additive explanations (SHAP) values. Red dots represent high values while blue dots represent low values. Dots extending toward the right of the center predict higher values for SBPSV.

Abbreviations: CKD, chronic kidney disease; CAD, coronary artery disease; DM, diabetes mellitus; ED, Emergency Department; EVD, external ventricular drain; HTN, hypertension; Hx, history; ICH, intracerebral hemorrhage; IV, intravenous; SHAP, Shapley Additive Explanations.

## Appendix D. Dot Plots from Random Forest Analysis for Features Predicting Standard Deviation in Systolic Blood Pressure During Emergency Department Stay

The Y-axis represents the order of significant contributions from top (most significant) to bottom (least significant). The X-axis represents the Shapley Additive Explanations (SHAP) values. Red dots represent high values while blue dots represent low values. Dots extending toward the right of the center predict higher values for SBPSD.

Abbreviations: CAD, coronary artery disease; DM, diabetes mellitus; ED, Emergency Department; EVD, external ventricular drain; HTN, hypertension; Hx, history; ICH, intracerebral hemorrhage; IV, intravenous; SHAP, Shapley Additive Explanations.

## Appendix F. Anderson–Darling’s Test

	**Anderson–Darling’s Test Value**	***p*-Value**
Age	0.613	0.109
Initial ED SBP	0.626	0.101
Initial ED heart rate	2	0.05
Sodium	1.9	0.05
ICH score	3.6	0.05
SBP upon leaving ED	0.95	0.16

While some variables appeared to have normal distributions on the histogram, their Anderson–Darling’s test value suggested otherwise. This was likely due to the small sample size.
